# Identification of new, emerging HIV-1 unique recombinant forms and drug resistant viruses circulating in Cameroon

**DOI:** 10.1186/1743-422X-8-185

**Published:** 2011-04-23

**Authors:** Viswanath Ragupathy, Jiangqin Zhao, Owen Wood, Shixing Tang, Sherwin Lee, Phillipe Nyambi, Indira Hewlett

**Affiliations:** 1 Lab of Molecular Virology, Center for Biologics Evaluation and Research, Food and Drug Administration, Bethesda, MD 20892, USA; 2 Department of Pathology, NYU School of Medicine, 550 First Avenue, Medical Sciences Building, 5th Floor, New York, NY 10016 423, USA

## Abstract

**Background:**

The HIV epidemic in Cameroon is characterized by a high degree of viral genetic diversity with circulating recombinant forms (CRFs) being predominant. The goal of our study was to determine recent trends in virus evolution and emergence of drug resistance in blood donors and HIV positive patients.

**Methodology:**

Blood specimens of 73 individuals were collected from three cities and a few villages in Cameroon and viruses were isolated by co-cultivation with PBMCs. Nested PCR was performed for gag p17 (670 bp) pol (840 bp) and Env gp41 (461 bp) genes. Sequences were phylogenetically analyzed using a reference set of sequences from the Los Alamos database.

**Results:**

Phylogenetic analysis based on partial sequences revealed that 65% (n = 48) of strains were CRF02_AG, 4% (n = 3) subtype F2, 1% each belonged to CRF06 (n = 1), CRF11 (n = 1), subtype G (n = 1), subtype D (n = 1), CRF22_01A1 (n = 1), and 26% (n = 18) were Unique Recombinant Forms (URFs). Most URFs contained CRF02_AG in one or two HIV gene fragments analyzed. Furthermore, pol sequences of 61 viruses revealed drug resistance in 55.5% of patients on therapy and 44% of drug naïve individuals in the RT and protease regions. Overall URFs that had a primary HIV subtype designation in the pol region showed higher HIV-1 p24 levels than other recombinant forms in cell culture based replication kinetics studies.

**Conclusions:**

Our results indicate that although CRF02_AG continues to be the predominant strain in Cameroon, phylogenetically the HIV epidemic is continuing to evolve as multiple recombinants of CRF02_AG and URFs were identified in the individuals studied. CRF02_AG recombinants that contained the pol region of a primary subtype showed higher replicative advantage than other variants. Identification of drug resistant strains in drug-naïve patients suggests that these viruses are being transmitted in the population studied. Our findings support the need for continued molecular surveillance in this region of West Central Africa and investigating impact of variants on diagnostics, viral load and drug resistance assays on an ongoing basis.

## Introduction

HIV/AIDS was first identified in Cameroon during 1985 [1] and the epidemic has continued to increase with the identification of multiple, divergent HIV subtypes and circulating recombinant forms (CRFs) [2]. According to a recent epidemiological surveillance report, 10,625 new infections were diagnosed in Cameroon during 2007 in comparison with 8,596 new infections during 2006 [3]. Furthermore, about 5.1% (ages 15-49) of adults are living with HIV/AIDS; among them, 60% (ages 15-49) were women. The majority of HIV infections in Cameroon are due to heterosexual transmission and high rates (40-50%) of infection have been observed among risk groups such as commercial sex workers and long distance truck drivers (UNAIDS/WHO) [4]. Antiretroviral therapy (ART) was initiated in Cameroon during 2001 and later decentralized to district level hospitals by the WHO 3by5 initiative (treating 3 million by 2005). In a study from Yaounde, Cameroon it was reported that 2.6% protease drug resistance and 9.3% major reverse transcriptase drug resistance were detected among patients who never received therapy, a finding that has implications for the efficacy of first line therapies [5]. Further in a study conducted at Doula, Cameroon [6] out of 819 patients who received first line ART, 36% had virological failure after 6 months or more. About 80% of drug resistance was detected for Nucleoside Reverse Transcriptase Inhibitors (NRTI) class, followed by the non-nucleoside reverse transcriptase Inhibitors (NNRTI) (76%) and Protease Inhibitor (PI) class (19%) drugs.

HIV infection in Cameroon is characterized by highly diversified strains including Circulatory Recombinant Forms (CRFs), Group O and N [7] which pose a challenge for diagnosis, vaccines and treatment [8]. Recently a new HIV strain, group P of gorilla origin, was identified in a Cameroonian woman [9] and shown to be distinct from other HIV groups O and N identified earlier in Cameroon [10,11]. Although new strains have been shown to emerge in Cameroon, studies that analyzed three immunodominant regions gag/pol/env have documented that 60-70% of infections continue to be CRF02_AG [12,13]. The current HIV molecular epidemic in Cameroon is predominantly based on CRF02_AG (65-75%), pure subtypes A1, A2, C, F2, G and H(1-5%), 6 different CRFs (-01, -11, -13, -18, -25, -37), divergent forms group O (2.2-3.8%) and HIV-2 (0.4-1.2%) [13-15]. Several previous reports on molecular epidemiology in Cameroon were from urban area using phylogenetic analysis of only gag and env gene sequences. In the first study it was reported that CRF02_AG accounted for 60%, followed by URFs(26%), 12 pure subtypes and CRFs [16] and in another study CRF02_AG accounted for 58.2% of infections followed by 14.8% of URFs, 0.2 - 6.1% of subtypes, A, B, C, D, F2, G and CRFs 01, 06, 09, 11, 13, 22, and 37 [12]. Both studies confirmed CRF02_AG is a dominant strain in urban Cameroon. However rural Cameroon comprises the majority of the country's population and in certain rural areas HIV prevalence has been found to be double the national rate (8-10%) [17]. In recent studies from rural Cameroon, CRF02_AG was shown to be a dominant strain 66.5% whether analyzed from a single pol fragment [14] or individual fragments of gag (65%), pol (75%) and env (55%) [13] followed by a second dominant strain CRF22_01_A1 in 5-10% of infections [14,18]. CRF22_01_A1 had been detected earlier [19] as a URF and later by full genome sequencing in two studies [14,18] and was found to be 2^nd ^dominant circulating strain in Cameroon. It is interesting to note that along with these subtypes and CRFs about 10-20% of strains were unique recombinant forms (URFs) that were likely generated by recombination of existing strains [12,19].

CRF02_AG recombinant viruses have become well established in the Cameroonian population possibly due to a founder effect from parent strains subtype A [20.21] and G [22] and/or a higher replicative advantage of CRF02_AG over other co-circulating recombinants [23,24]. A prospective study confirmed that CRF02_AG is the predominant strain in blood donors in Yaoundé, Cameroon [12]; however, other CRFs and Unique Recombinant Forms (URFs) have also been detected [25-27]. The ongoing evolution of HIV and emergence of new recombinant forms are a major concern for the global AIDS pandemic. Several factors may contribute to the emergence of recombinant viruses or URFs but most importantly, recombination provides a mechanism to increase viral sequence diversity rapidly, unlike the slow accumulation of mutations that occurs through replication errors [28]. For recombination to occur between distinct HIV-1 strains, a cell needs to be dually infected with different viruses the progeny virions that result possess RNA genomes from each virus, permitting strand-switching to occur during the next round of reverse transcription. Therefore, recombination requires co-infection of viral strains in an individual. This dual infection may occur during the primary infection period, before the immune response is fully developed, or it may occur as a superinfection with a new viral strain after the initial strain has established a chronic infection. Super infection with a different strain is thought to be the most predominant contributor to viral recombination [29]. The possibility for superinfection among high risk individuals has been reported in Cameroon [30]. Both superinfection and recombination have the potential to complicate efforts to develop vaccines, and reinfection with a drug-resistant virus could jeopardize available treatment options. To date 49 CRFs have been identified [31] and recombination between the URFs and existing CRFs results in Second Generation Recombinants (SGRs) [27] which would further complicate the phylogenetic nature of the epidemic. In the present study we have analyzed recent trends in the genetic evolution of the HIV epidemic in different regions of Cameroon and demonstrated the ongoing emergence of new recombinants and prevalence of multi-drug resistant viruses in the population.

## Materials and methods

### Study Population

**Figure 1:**
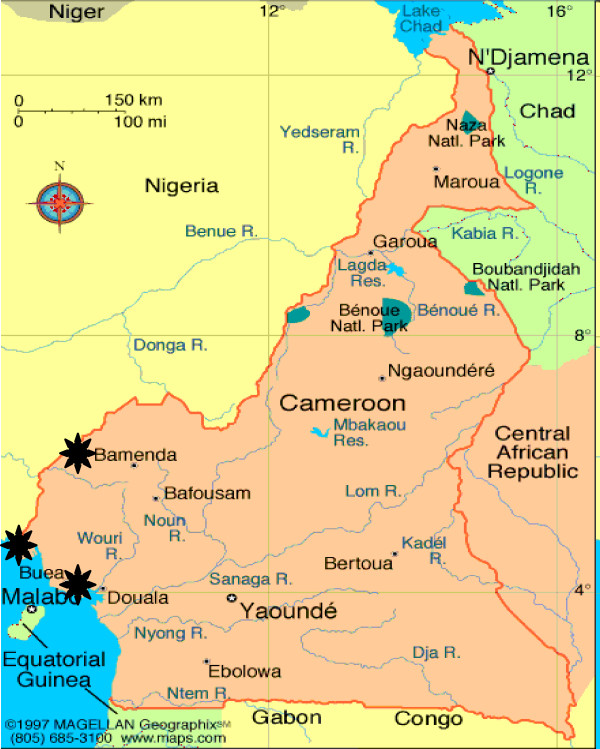
Samples for this study were collected from Bamenda, Buea and Limbe in Cameroon are indicated with an asterisk (*).

Blood samples were received from blood banks and clinics in 3 cities in Cameroon (Figure 1), samples from blood banks were from Bamenda (ARC) (n = 28) in the North West and other clinical sites at Buea (BDHS) (n = 21), Limbe (LPH) (n = 16) in the South West and a few villages in Cameroon (NYU) (n = 8). Demographic information was collected in the performa. The specimens used for this study had all been previously obtained for another purpose and were de-identified for the analysis.

### Virus culture, polymerase chain reaction (PCR) and sequencing

Seventy three viruses were cultured in peripheral blood mononuclear cells (PBMCs) of buffy coat received from HIV seronegative blood donors from the NIH Blood bank. Viruses were propagated as per our standard protocol of the laboratory published earlier [32]. Cell free viruses were harvested and stored frozen at -80°C. Viral RNA was isolated using the QIAGEN viral RNA kit (Cat: 52906) and complementary DNA was synthesized in a 20 ul reaction using Invitrogen Superscript III RT kit (Cat: 18080-051) and custom primers BOA (HXB2 coordinate 5242-5267) and DOAR (HXB2 coordinate 9513-9538). Briefly, reaction conditions were 65°C for 5 min with template and 10 mM dNTP mix, followed by 50°C for 50 min with RT reagents and two fragments of cDNA was synthesized from the template RNA. Nested PCR reaction was performed for Gp41 (461 bp), p17 (670 bp) of all 73 isolates and 61 viruses for pol (840 bp) region. The reaction mixture consisted of PCR pre mix buffer from Invitrogen (Cat No: M7505), 25 pmole of each primer (Table 1) for both the rounds of PCR and reaction conditions were one cycle at 94°C for 3 min, 30 cycles at 94°C for 30 s, 50°C for 30 s, 72°C for 1.2 min and final extension 72°C for 7 minutes. Amplified products were detected by 1% agarose gel electrophoresis. All PCR fragments were sequenced by ABI dye terminator reaction systems.

**Table 1:** List of PCR primers used for amplification of gp41, p17 and pol genes of HIV.

Gene	Primer	Reaction	Sequence	HXB2 coordinates	Size in bp
gp41*	gp40F1 gp41R1	1^st ^PCR	*TCTTAGGAGCAGCAGGAAGCACTATGGG* *AACGACAAAGGTGAGTATCCCTGCCTAA*	7840-8300	461
	gp46F2 gp47R1	2^nd ^PCR	*ACAATTATTGTCTGGTATAGTGCAACAGCA* *TTAAACCTATCAAGCCTCCTACTATCATTA*		

p17*	pL393 pL392	1^st ^PCR	*AAGGGTACTAGTAGTTCCTGCTATG* *GCTGAAGCGCGCACGGCAAGAG*	761-1437	670
	p17-1033 p17-1048	2^nd ^PCR	*TCTATCCCATTCTGCAGCTTCCTCATTGAT* *TTTGACTAGCGGAGGCTAGA*		

pol	ANA corr SP5R	1^st ^PCR	*CAGGAGCAGATGATACAGTATTAG* *ATTTATCAGGATGGAGTTCA*	2390-3229	840
	AOA Corr SP5F-r	2^nd ^PCR	*GATAGGGGGAATTGGAGG* *AATGGAGGTTCTTTCTGATGT*		

* These primer sets were shown to amplify nucleic acid from Cameroonian viruses efficiently in previous studies [13].

### Sequence Analysis

A phylogenetic analysis was performed using partial genome segments and analyzed by the MEGA 4.1 software package. Pair wise evolutionary distances were generated using Kimura's two-parameter method; major gaps in the alignment were masked out prior to analysis and phylogenetic trees were constructed by neighbor-joining [33,34]. Nucleotide sequences were aligned by the Clustal W program [34] using partial curated alignments of 42 CRFs and inter-subtypes as reference sequences. All reference subtypes and CRFs were obtained from the Los Alamos HIV Sequence Database and initially used to construct the trees [31]. Some references have been omitted during the analysis for clarity and all positions with alignment gaps were removed. Confidence values for individual branches have been determined using bootstrap analysis.

The 840 bp (Hxb2 position 2390-3229) genomic region studied for reverse transcriptase (codon 1-227) and protease drug (codon 46-99) resistance may not represent the entire profile of drug associated mutations. However this paper analyzed 80% of the protease and the entire RT region of the pol gene. HIV drug resistance was analyzed using the Stanford University HIValg Program [compares three programs HIVdb, Rega Institute and Agence Nationale de Recherches surle SIDA (ANRS)]. The 61 pol sequences were analyzed for mutations associated with NRTI, NNRTI and PI drug resistance using the HIValg program available on the Stanford University HIV Drug Resistance Database website [35]. Mutations in the sequences were defined as differences from the consensus B reference sequence and were further characterized as NRTI-resistance mutations, NNRTI-resistance mutations, PI-resistance mutations or other mutations which consists primarily of accessory mutations (those which alone or have little or no effect on drug susceptibility) and polymorphisms. For each sequence analyzed, the drug resistance interpretation was compared for consistency and only consistent mutations in 2 or more algorithms predicted are presented in the Table. Since data generated from 61 viruses was too large for inclusion in the manuscript it has been summarized in the form of a Table (data sheets may be provided for individual requests).

### Sequence Information

The sequences generated for this study are available from GenBank under the accession numbers for p17 (FJ014614-FJ014646); gp41 (FJ014673-FJ014703); pol (FJ014647-FJ014672).

## Results

The demographics in terms of gender, age and route of HIV acquisition are outlined below. Seventy three samples obtained from 59 females and 14 males were analyzed in this study. The ages of men and women were 35-55 and 16-59 years respectively. Heterosexual contact accounted for 94% of infections and 6% reported having no extramarital relations but had received blood transfusions. Out of 61 patients, 18 (29%, 11 female and 7 male) reported having received therapy (3by5 initiated by WHO); the other 43 (71%, 5 men and 38 women) were drug naïve.

**Figure 2:**
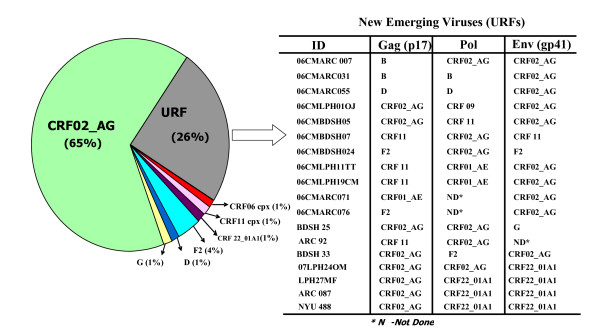
**The Pie diagram shows the distribution of pure HIV subtypes and URFs**. Discordant subtypes of URFs in the gag (p17), pol and Env (gp41) are indicated in the table.

Out of 73 samples, PCR was successful for 72 samples in the p17 (gag), 70 samples in the gp41 (env) regions and 61 samples in the pol region. Genotyping analysis of the p17 region revealed that 75% were CRF0_2AG, 7% were CRF11 and F2, 3% were subtype CRF22_01A1, D, B and 1% was CRF06, and subtype G (Table 2). The pol region analysis revealed that 74% were CRF02_AG, 5% were subtype F2 and CRF22_01A1, 3% were CRF01_AE and subtype D, 2% were comprised of CRF06, CRF 11, CRF09, B and G (Table 2). Gp41 genotyping revealed that 79% were CRF02_AG, 6% were subtype F2, 7% were CRF22_01A1, 3% were CRF11 and G, 1% was subtype D and CRF06 (Table 2). Based on sequence analysis of 2 or 3 distinct genes, HIV genotypes circulating in Cameroon were found to be CRF02_AG (65%), URFs (26%) subtype F2 (4%) and 1% each of CRF06, CRF11, CRF22_01A1, subtype G and D (Figure 2). Phylogenetic analyses are assessed from enclosed additional file 1.

**Table 2:** Partial Genotypes based on p17/pol/gp41 genes of HIV for samples from Cameroon

Sample ID	Gag(p17)	pol	Env(gp41)	Genotype
06CMARC009	CRF02	CRF02	CRF02	CRF02_AG
06CMARC010	CRF02	CRF02	CRF02	CRF02_AG
06CMARC011	CRF02	CRF02	CRF02	CRF02_AG
06CMARC023	CRF02	CRF02	CRF02	CRF02_AG
06CMARC036	CRF02	CRF02	CRF02	CRF02_AG
06CMARC053	CRF02	CRF02	CRF02	CRF02_AG
06CMARC058	CRF02	CRF02	CRF02	CRF02_AG
06CMLPH016SL	CRF02	CRF02	CRF02	CRF02_AG
06CMLPH17HT	CRF02	CRF02	CRF02	CRF02_AG
06CMLPH03VJ	CRF02	CRF02	Not Done	CRF02_AG
06CMLPH22NH	CRF02	CRF02	CRF02	CRF02_AG
06CMLPH20SL	CRF02	CRF02	CRF02	CRF02_AG
06CMLPH02MG	CRF02	CRF02	CRF02	CRF02_AG
06CMLPH05DE	CRF02	CRF02	CRF02	CRF02_AG
07CMLPH128	CRF02	CRF02	Not Done	CRF02_AG
06CMARC001	CRF02	Not done	CRF02	CRF02_AG
06CMARC004	CRF02	Not done	CRF02	CRF02_AG
06CMARC006	CRF02	Not done	CRF02	CRF02_AG
06CMARC065	CRF02	Not done	CRF02	CRF02_AG
06CMARC066	CRF02	Not done	CRF02	CRF02_AG
06CMARC067	CRF02	Not done	CRF02	CRF02_AG
06CMARC068	CRF02	Not done	CRF02	CRF02_AG
06CMARC069	CRF02	Not done	CRF02	CRF02_AG
BDHS 131	CRF02	CRF02	CRF02	CRF02_AG
BDHS 139	CRF02	CRF02	CRF02	CRF02_AG
NYU 707	CRF02	CRF02	CRF02	CRF02_AG
NYU 871	CRF02	CRF02	CRF02	CRF02_AG
NYU 997	CRF02	CRF02	CRF02	CRF02_AG
NYU 1003	CRF02	CRF02	CRF02	CRF02_AG
ARC 152	CRF02	CRF02	CRF02	CRF02_AG
ARC 155	CRF02	CRF02	CRF02	CRF02_AG
ARC 097	CRF02	CRF02	CRF02	CRF02_AG
BDHS 09	CRF02	CRF02	CRF02	CRF02_AG
BDHS12	CRF02	CRF02	CRF02	CRF02_AG
ARC 88	CRF02	CRF02	CRF02	CRF02_AG
ARC 140	CRF02	CRF02	CRF02	CRF02_AG
06BDHS18	CRF02	CRF02	CRF02	CRF02_AG
BDHS 28	CRF02	CRF02	CRF02	CRF02_AG
BDHS 35	CRF02	CRF02	CRF02	CRF02_AG
BDHS 52	Not done	CRF02	CRF02	CRF02_AG
BDHS 56	CRF02	CRF02	CRF02	CRF02_AG
LPH21MJ	CRF02	CRF02	CRF02	CRF02_AG
NYU 474	CRF02	CRF02	CRF02	CRF02_AG
LPH09OR	CRF02	CRF02	CRF02	CRF02_AG
NYU 477	CRF02	Not done	CRF02	CRF02_AG
NYU 807	CRF02	CRF02	CRF02	CRF02_AG
07CMBDHS064	CRF02	CRF02	CRF02	CRF02_AG
06CMARC013	CRF06	CRF06	CRF06	CRF06 cpx
BDHS 138	CRF22_01A1	CRF22_01A1	CRF22_01A1	CRF22_01A1
07BDHS 018	CRF 11	Not done	CRF 11cpx	CRF 11 cpx
BDHS 132	F2	F2	F2	F2
06CMBDHS019	F2	F2	F2	F2
BDHS 36	F2	Not done	F2	F2
BDHS 13	D	D	D	D
LPH28AF	G	G	G	G
LPH24OM	CRF02	CRF02	CRF22_01A1	URF
06CMARC007	B	CRF02	CRF02	URF
ARC 92	CRF 11	CRF02	Not Done	URF
06CMARC031	B	B	CRF02	URF
06CMARC055	D	D	CRF02	URF
06CMLPH01OJ	CRF02	CRF09	CRF02	URF
06CMBDHS05	CRF02	CRF11	CRF02	URF
06CMBDHS07	CRF 11	CRF02	CRF 11	URF
06CMBDHS024	F2	CRF02	F2	URF
06CMLPH11TT	CRF 11	CRF01_AE	CRF02	URF
06CMLPH19CM	CRF 11	CRF01_AE	CRF02	URF
06CMARC071	CRF22_01A1	Not done	CRF02	URF
06CMARC076	F2	Not done	CRF02	URF
BDHS 25	CRF02	CRF02	G	URF
NYU 488	CRF02	CRF02	CRF22_01A1	URF
BDHS 33	CRF02	F2	CRF02	URF
LPH27MF	CRF02	CRF22_01A1	CRF22_01A1	URF
ARC 87	CRF02	CRF22_01A1	CRF22_01A1	URF

**Figure 3:**
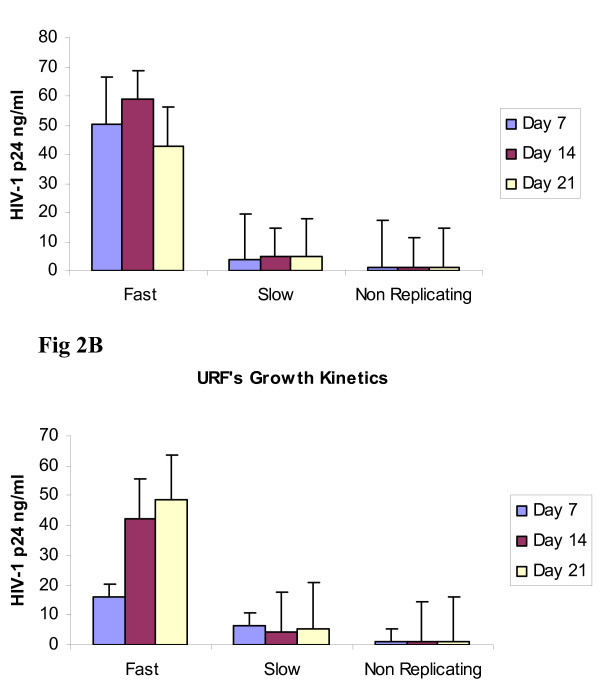
**Figure 3A and 3B are histogram representations of the average HIV-1 p24 levels of CRF02_AG and URFs in cell culture studies**.

The replication kinetics of CRF02_AGs and URF strains were classified as fast, slow and no growth viruses. For the analysis, viruses that produced concentrations of HIV p24 >10-100 ng/ml at Day 7 were classified as fast growing viruses and those <10 or 0 ng/ml as slow or non replicating viruses. In the present study, p24 antigen levels in CRF02_AG cultures were 50.5 ng/ml at day 7 (Figure 3A) while levels in CRF02_AG containing URFs were 16.1 ng/ml (Figure 3B). Slow viruses had 3.6 to 0.1 ng/ml of p24 levels in the culture supernatants at day 7. Further more, slow growing viruses showed an increase of 1-2 ng of p24 at day 14 and 21, however some viruses showed no p24 increases during the culture period and hence were classified as non replicating viruses.

Of 61 viruses sequenced for HIV-1 drug resistance mutations, 18 had received antiretroviral therapy and 43 were drug naïve. Among the group that received antiretroviral therapy (ART), 8 viruses (44%) showed no drug-specific mutations, while 10 (55.5%) had drug-specific mutations in the RT and protease regions. Two ART patients harbored viruses with multi drug resistance for NNRTI/NRTI and 3 had low to high level mutations for NRTI/NNRTI. Interestingly 44% (19/43) of drug naïve patients were found to have one or more drug specific mutations in the RT and protease regions. However, only 42% (8/19) of mutations were those that confer drug resistance (Table 3), and 26% (11/43) of mutations were polymorphisms in the RT region. The impact of these polymorphisms for effective therapy of these patients is unknown.

**Table 3:** Drug sensitivity and resistance mutations in the reverse transcriptase and protease regions.

Sample ID	ARV Therapy	RT Resistance	Protease Resistance	Genotype
06CMARC010	No	Sensitive	Sensitive	CRF02_AG
06CMARC011	Yes	--	--	CRF02_AG
06CMARC031	Yes	--	--	URF
06CMARC053	Yes	--	--	CRF02_AG
06CMARC055	Yes	--	--	URF
06CMARC058	Yes	--	--	CRF02_AG
06CMLPH02MG	No	--	--	CRF02_AG
06CMLPH05DE	No	--	--	CRF02_AG
06CMLPH03VJ	No	--	--	CRF02_AG
06CMLPH11TT	No	--	--	URF
06CLPH16SL	Yes	--	--	CRF02_AG
07CMLPH128	Yes	--	--	CRF02_AG
06CMBDHS07	Yes	--	--	URF
06CMLPH22NH	No	--	--	CRF02_AG
06CMLPH01OJ	No	--	--	URF
NYU 707	No	--	--	CRF02_AG
ARC 155	No	--	--	CRF02_AG
BDHS12	No	--	--	CRF02_AG
06BDHS18	No	--	--	CRF02_AG
BDHS 28	No	--	--	CRF02_AG
LPH21MJ	No	--	--	CRF02_AG
LPH24OM	No	--	--	URF
ARC 097	No	--	--	CRF02_AG
ARC 93	No	--	--	CRF02_AG
BDHS 25	No	--	--	URF
NYU 807	No	--	--	CRF02_AG
NYU 474	No	--	--	CRF02_AG
NYU 488	No	--	--	URF
LPH27MF	No	--	--	URF
BDHS 132	No	--	--	F2
BDHS 13	No	--	--	D
LPH28AF	No	--	--	G

**06CMBDHS05**	**Yes**	**A62V, V75I, F77L, F116Y, Q151M, M184V, Y188L** **A62V, K65R, T69I, V75I, V108I, F116Y, Q151M**,	**--**	**URF**
**06CMARC007**	**Yes**	**Y181C, M184I**	**--**	**URF**
**06CMARC036**	**Yes**	**K103N, V179E, Y181C, M184V, G190A**	**--**	**CRF02_AG**
**06CMARC009**	**Yes**	**V179E**	**--**	**CRF02_AG**
**06CMLPH17HT**	**Yes**	**G190A**	**--**	**CRF02_AG**
**07CMBDHS064**	**Yes**	**G190A**	**--**	**CRF02_AG**
**06CMARC023**	**Yes**	**L100Q, V108I**	**--**	**CRF02_AG**
**ARC 88**	**Yes**	**D67N, T69N, K70R, L100I, K103N, M184V**	**--**	**CRF02_AG**
**ARC 140**	**Yes**	**D67N, T69N, K70R, L100I, K103N, M184V**	**--**	**CRF02_AG**
**ARC 87**	**Yes**	**T69I**	**--**	**URF**

**06CMLPH20SL**	**No**	**V108I, F116Y, Q151M, Y181C, M184V**	**--**	**CRF02_AG**
**LPH 09OR**	**No**	**K219Q**	**--**	**CRF02_AG**
**ARC 152**	**No**	**V179E**	**--**	**CRF02_AG**
**06CMLPH19CM**	**No**	**V118I**	**--**	**URF**
**06CMBDHS024**	**No**	**G190A**	**--**	**URF**
**06CMBDHS019**	**No**	**V108I, Y181C, M184V**	**--**	**F2**
**06CMARC013**	**No**	**V179E**	**--**	**CRF06_cpx**
**BDHS 52**	**No**	**H221K**	**I54L**	**CRF02_AG**

**BDHS 35**	**No**	**V106L + D67H, V75L**	**50S**	**CRF02_AG**
**BDHS 56**	**No**	**K219R**	**--**	**CRF02_AG**
**BDHS 131**	**No**	**V90I**	**--**	**CRF02_AG**
**BDHS 139**	**No**	**Y188F, Q151P, L210P**	**--**	**CRF02_AG**
**NYU 871**	**No**	**V90I**	**--**	**CRF02_AG**
**NYU 997**	**No**	**T215A**	**--**	**CRF02_AG**
**NYU 1003**	**No**	**V106I**	**--**	**CRF02_AG**
**BDHS 09**	**No**	**L100I, Y181I**	**--**	**CRF02_AG**
**ARC 92**	**No**	**Q151R, V179D, G190R**	**--**	**URF**
**BDHS 33**	**No**	**E44D, K19T, H221K**	**--**	**URF**
**BDHS 138**	**No**	**T69I, l74f, A98G, E138A**	**--**	**CRF22_01A1**

Drug resistance mutation detected in patients on therapy and drug naïve are highlighted bold. Patient samples having polymorphisms at drug resistance codons are highlighted bold in last section.

Among persons who showed primary drug resistance mutations, one patient (06CMLPH20SL) had multi drug resistance mutations for NNRTI/NRTI; two other patients (BDSH 35 and BDSH 52) had protease drug point mutations of I54L, which confers low level resistance for several protease drugs except tipranavir, and I50S, which is a polymorphism (Table 3). Furthermore, these individuals had NNRTI associated mutations in H221K/V106L/D67H and V75L, however these mutations were classified as polymorphisms. Three other patient viruses had point mutations for EFV/DLV/3TC/NVP or Etravirine and two patients had the K219R/Q Zidovudine (AZT) point mutation (Table 3).

Since significant percentages (26%) of viral strains were found to be URFs, we analyzed the three genomic regions of HIV independently to determine phylogenetic associations between the URFs and other strains in the regions studied. Interestingly, two pairs of p17/pol/gp41 sequences phylogenetically clustered with high bootstrap 97-99% values (Table 4). One pair of sequences (06CMLPH19CM; 06CMLPH11TT) clustered 99% in the p17/pol/gp41. However, a second pair (06CMBDHS024; 06CMBDHS019) clustered only in the p17 and gp41 region. The pol gene of 06CMBDHS024 clustered with the pol gene of 06CMLPH17HT. Since both patients had distinct genotypes, the resulting genotype of 06CMBDHS024 was a URF (Table 4). Such associations were not observed with partial sequences from other patients analyzed.

**Table 4:** Phylogenetic association of patient isolates.

Sample ID	Sex	Mode of Transmission	Place	Bootstrap	Gag-p17	Pol	Env-gp41	Genotype
**Gag Sequences (p17)**								
06CMBDHS019	F	Hx.Sex	Buea	100%	F2	F2	F2	F2
06CMBDHS024	F	Hx.Sex	Buea		F2	CRF02	F2	URF*

06CMLPH11TT	F	Hx. Sex	Limbe	100%	CRF11	CRF01_AE	CRF02	URF
06CMLPH19CM	F	Hx. Sex	Limbe		CRF11	CRF01_AE	CRF02	URF

**Pol Sequences**								
06CMLPH19CM	F	Hx. Sex	Limbe	99%	CRF11	CRF01_AE	CRF02	URF
06CMLPH11TT	F	Hx. Sex	Limbe		CRF11	CRF01_AE	CRF02	URF

06CMLPH17HT	M	Hx. Sex	Limbe	99%	CRF02	CRF02	CRF02	CRF02
06CMBDHS024	F	Hx.Sex	Buea		F2	CRF02	F2	URF*

**Env Sequences (gp41)**								
06CMLPH02MG	F	Hx. Sex	Limbe	97-99%	CRF02	CRF02	CRF02	CRF02
06CMLPH17HT	M	Hx. Sex	Limbe		CRF02	CRF02	CRF02	CRF02

06CMBDHS019	F	Hx. Sex	Buea	99%	F2	F2	F2	F2
06CMBDHS024	F	Hx.Sex	Buea		F2	CRF02	F2	URF*

Phylogenetically associated sequences are in rows for p17, pol and Env gp41 with Bootstrap values indicated. Columns indicate demographics and genotype. Patient IDs marked (*) demonstrate the emergence of URF’s within the population or that potentially had a common source of infection.

Hx: Heterosexual, *pol gene has CRF02_AG

## Discussion

The present study reveals the ongoing genetic diversity of HIV-1 in three cities Bamenda in the Northwest and Buea and Limbe in the Southern provinces of Cameroon based on genotyping of samples collected from 2006-2008. Even though these samples may not reflect the entire Cameroon population this study highlights the importance and dynamic nature of HIV evolution in this region. Viral diversity and drug resistance was observed in samples from both blood banks and other clinical sites. Subtype determination based on nucleotide sequence analysis of three distinct regions of the HIV-1 genome provides more detailed information of the phylogenetics of the HIV-1 epidemic in a specific region. In this study 66% of patients and blood donors studied harbored the CRF02_AG strain. Similar studies conducted on samples collected during the early part of the past decade showed that CRF02_AG was dominant in blood donors in Cameroon along with other HIV subtypes and CRFs [36]. It has been reported that CRF02_AG appeared to be stable in blood donors in Yaounde over the period of 1996-2004 [12] and this strain may have a higher fitness than other strains circulating in the country with high viral load and higher infectivity over other forms of HIV [37]. It is interesting to note that 26% of strains were URFs of CRF02_AG with existing subtypes F, B, D, G and CRF 11, CRF 06. Circulation of such HIV variants may be a factor in the evolution of the phylogenetic nature of the epidemic in this geographic region. Further longitudinal studies are needed in these areas to monitor HIV evolution, virus genetic, diversity and its immunologic impact. Host genetic factors that may contribute to the genetic diversity in this region may also be worthy of investigation and could provide new insights into HIV evolution. As noted earlier, the expanding genetic diversity raises public health concerns including the ability of diagnostic assays to detect these unique HIV mosaic variants.

In an earlier study [38] it was observed that a small number of CRFs yielded discordant results with licensed assays, an observation consistent even with the plasma samples collected during 2007-2009 (unpublished data) highlighting the need for continued monitoring of variants and their impact on diagnostic assay sensitivity.

### Growth Kinetics

The replication characteristics of the viruses were examined by performing co-cultivation studies with primary PBMCs. Viruses were classified as fast, slow and non replicating viruses. The majority of the viruses yielded high levels of HIV p24 antigen in cell culture, however some viruses yielded low levels or did not replicate. Most importantly, in our study we observed high levels of p24 antigen production with recombinants of CRF02_AG that contained URFs of the pol region of CRF01_AE, CRF_ 22 or subtype D when compared with recombinants that had a primary subtype F2 with CRF02_AG in the pol region. These findings suggest that the generation of new recombinants may be accompanied by variations in replication characteristics of the new strains which in turn may have an impact on virus transmission in this population.

### Drug Resistance

Our next effort was to determine whether the available therapeutic options could be affected by emerging recombinant HIV strains. Among the 29% of patients that received ART, 55.5% had drug specific mutations. Most patients received NRTI/NNRTI standard combinations for the duration of 1-2 years at the time of sampling and very few received single dose Nevirapine or AZT to reduce parent-to-child transmission. Four patients (06CMBDSH05, 06CMARC007, ARC88 and ARC140) had resistance for all classes of RT antiretroviral therapy (Table 3) and the partial genotype of these isolates was CRF02_AG that contained URFs. Another two individuals (06CMLPH20SL and 06CMBDSH019) were on standard ART regimens available in Cameroon. Drug resistance profiles of the Cameroonian population from earlier studies had shown the presence of drug resistance associated with lamivudine efavirenz, and nevirapine [39,40]. These drugs are widely used in Cameroon in either individual formulations or fixed-dose combinations. The fixed-dose combination of lamivudine/stavudine/nevirapine is now the most frequently prescribed drug in Cameroon and other African countries [41]. Since these resistance mutations were detected among patients with diverse strains we hypothesize that drug-related selection pressures may also be a contributing factor for viral evolution.

Other NNRTI single mutations detected among patients on therapy include G190A, V118I, V179E, K219Q and H221K and these unusual mutations may be polymorphisms for the CRF02_AG or CRF02_AG containing URFs. Follow up studies are needed to provide more information on the implications of these mutations for pathogenesis and treatment. However, it has been reported that G190A mutation alone would decrease the susceptibility to nevirapine [42] and its presence as polymorphisms would impact prevention measures for mother-to-child transmission based on the drug.

Interestingly 19/43 (44%) of drug naïve patients had drug resistance or polymorphisms at drug resistance sites. These findings suggest that resistant viruses were being transmitted within the study group. Detailed analysis of resistant codons (Table 3) 8/19 (42%) showed RTI associated mutations. Among drug naïve patients, one patient (06CMLPH20SL) had multidrug resistance for all classes of RTIs and another 6 patients had mutations that confer resistance to RT and protease drugs (Table 3). Such individuals pose a major concern for antiretroviral therapies provided by public health prevention programs in the country. These studies also highlight the importance of drug resistance testing prior to initiation of therapy.

In the present study two individuals (Table 3) had point mutations for protease drug who had never received these drugs. A mutation at I54L reduces the susceptibility for fosamprenavir (FPV), lopinavir (LPV) and darunavir (DRV) but not Tipranavir (TPV). These mutations mostly occur in patients receiving these drugs but their presence as polymorphisms in patients with CRF02_AG genotype limits future use of these drugs. In Cameroon these mutations appear to be prevalent among drug naïve individuals [43,44]. These observations suggest that they would have been transmitted with mutations for protease drug or that CRF02_AG also had natural polymorphism at these positions.

All results were from random sampling at one single time point. In the future, linked studies will be required to determine the importance of emerging drug resistance in the regions studied.

### Genetic variation and phylogenetic relatedness

The identification of divergent recombinant strains in three cities in Cameroon is consistent with findings reported by earlier investigators, we wished to examine whether specific segments of the HIV genome are phylogenetically associated with high bootstrap values within the population. Analysis of sequence similarity within the population in the p17/pol/gp41 region of two pairs of sequences showed that in each of them p17/pol/gp41 were associated in only 2 of the 3 genomic regions. The bootstrap values for these associations were 97-100% similar to values observed in direct transmission cases such as intravenous drug users [45] or mother to child transmission [46] In our study we observed high bootstrap values for some viruses suggesting that perhaps such associations may exist in specific cases. In particular one isolate (06CMBDHS024) that was primarily subtype F2 contained a pol segment of CRF02_AG, and when further analyzed this virus was closely associated in the pol region with the second isolate (06CMLPH17HT). These findings suggest epidemiological associations between them and it should also be noted that these two isolates were from two different cities. Extensive molecular epidemiological analysis linked with behavioral studies may provide a new approach for determining the public health significance of the emergence of new URFs in regions where multiple strains circulate.

In conclusion, our study suggests that the genetic diversity of the HIV epidemic in Cameroon is evolving and periodic monitoring of HIV variants is necessary to determine the extent of virus evolution in this region. These studies are important as strains identified first in Cameroon and West Central Africa including CRF02_AG and CRF01_AE have eventually been transported and become prevalent in other parts of the world through globalization. It is also necessary to study genetic variation and understand its impact on licensed diagnostic and nucleic acid monitoring tests. Finally, the identification of drug resistance in drug naïve patients highlights the need to monitor viral genotype before treatment and after ART failure. A comprehensive genetic analysis of, and surveillance for, emerging HIV variants in major cities and rural areas on a periodic basis is clearly warranted in these regions of world. The insights gained from these studies will help us identify diagnostic, therapy and vaccine strategies associated with the discoveries of new strains that could successfully bolster the fight ongoing against HIV/AIDS.

## Competing interests

The authors certify that there is no conflict of interest with any financial organization regarding the material discussed in the manuscript.

## Authors' contributions

VR drafted the manuscript and made substantial contributions to conception, design, analysis and interpretation of data, JZ helped with sequence data analysis, OW cultured viruses for this study, ST provided suggestions for the work and reviewed the manuscript, SL assisted with p24 analysis, PN provided the viruses for the work, IH was involved in conception and supervision of the project and provided input in revising the manuscript critically for publication. All authors read and approved the final manuscript

## Supplementary Material

Additional file 1 **Tree file**. This document contains phylogenetic tree files for p17 (gag), pol and gp41 (env) regions of HIV. Patient isolates were placed after red dot. The Subtype with which Cameroon viruses clustered was indicated in the right side with corresponding arrow mark. Each of gag (2a1-a3),pol (2b1-b3) and env (2c1-c3) fragments has 3 trees. Click here for file
