# Supplementary material for: Identification of new, emerging HIV-1 unique recombinant forms and drug resistant viruses circulating in Cameroon
Source: Virol J. 2011 Apr 23;8:185. doi: 10.1186/1743-422X-8-185 (PMC3118203; doi:10.1186/1743-422X-8-185)
Supplement: Additional file 1 — Tree file. This document contains phylogenetic tree files for p17 (gag), pol and gp41 (env) regions of HIV. Patient isolates were placed after red dot. The Subtype with which Cameroon viruses clustered was indicated in the right side with corresponding arrow mark. Each of gag (2a1-a3),pol (2b1-b3) and env (2c1-c3) fragments has 3 trees. [file 1743-422X-8-185-S1.PDF]

Supporting phylogenetic tree files for p17 (gag), pol and gp41 (env) regions of HIV

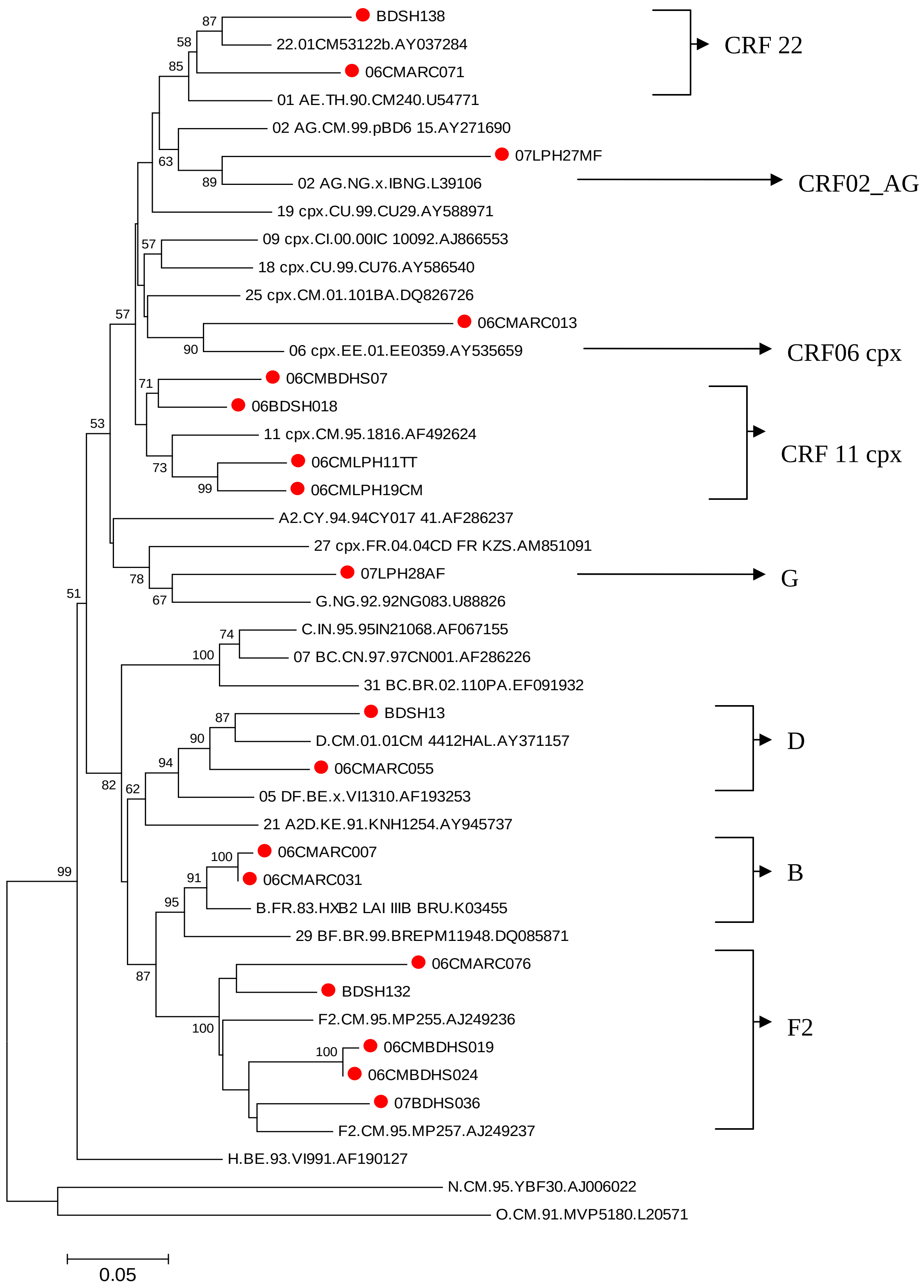
BDSH138
22.01CM53122b.AY037284
06CMARC071
01 AE.TH.90.CM240.U54771
02 AG.CM.99.pBD6 15.AY271690
07LPH27MF
02 AG.NG.x.IBNG.L39106
19 cpx.CU.99.CU29.AY588971
09 cpx.CI.00.00IC 10092.AJ866553
18 cpx.CU.99.CU76.AY586540
25 cpx.CM.01.101BA.DQ826726
06CMARC013
06 cpx.EE.01.EE0359.AY535659
06CMBDHS07
06BDSH018
11 cpx.CM.95.1816.AF492624
06CMLPH11TT
06CMLPH19CM
A2.CY.94.94CY017 41.AF286237
27 cpx.FR.04.04CD FR KZS.AM851091
07LPH28AF
G.NG.92.92NG083.U88826
C.IN.95.95IN21068.AF067155
07 BC.CN.97.97CN001.AF286226
31 BC.BR.02.110PA.EF091932
BDSH13
D.CM.01.01CM 4412HAL.AY371157
06CMARC055
05 DF.BE.x.VI1310.AF193253
21 A2D.KE.91.KNH1254.AY945737
06CMARC007
06CMARC031
B.FR.83.HXB2 LAI IIIB BRU.K03455
29 BF.BR.99.BREPM11948.DQ085871
06CMARC076
BDSH132
F2.CM.95.MP255.AJ249236
06CMBDHS019
06CMBDHS024
07BDHS036
F2.CM.95.MP257.AJ249237
H.BE.93.VI991.AF190127
N.CM.95.YBF30.AJ006022
O.CM.91.MVP5180.L20571
CRF 22
CRF02_AG
CRF06 cpx
CRF 11 cpx
G
D
B
F2
0.05

Fig 2a1

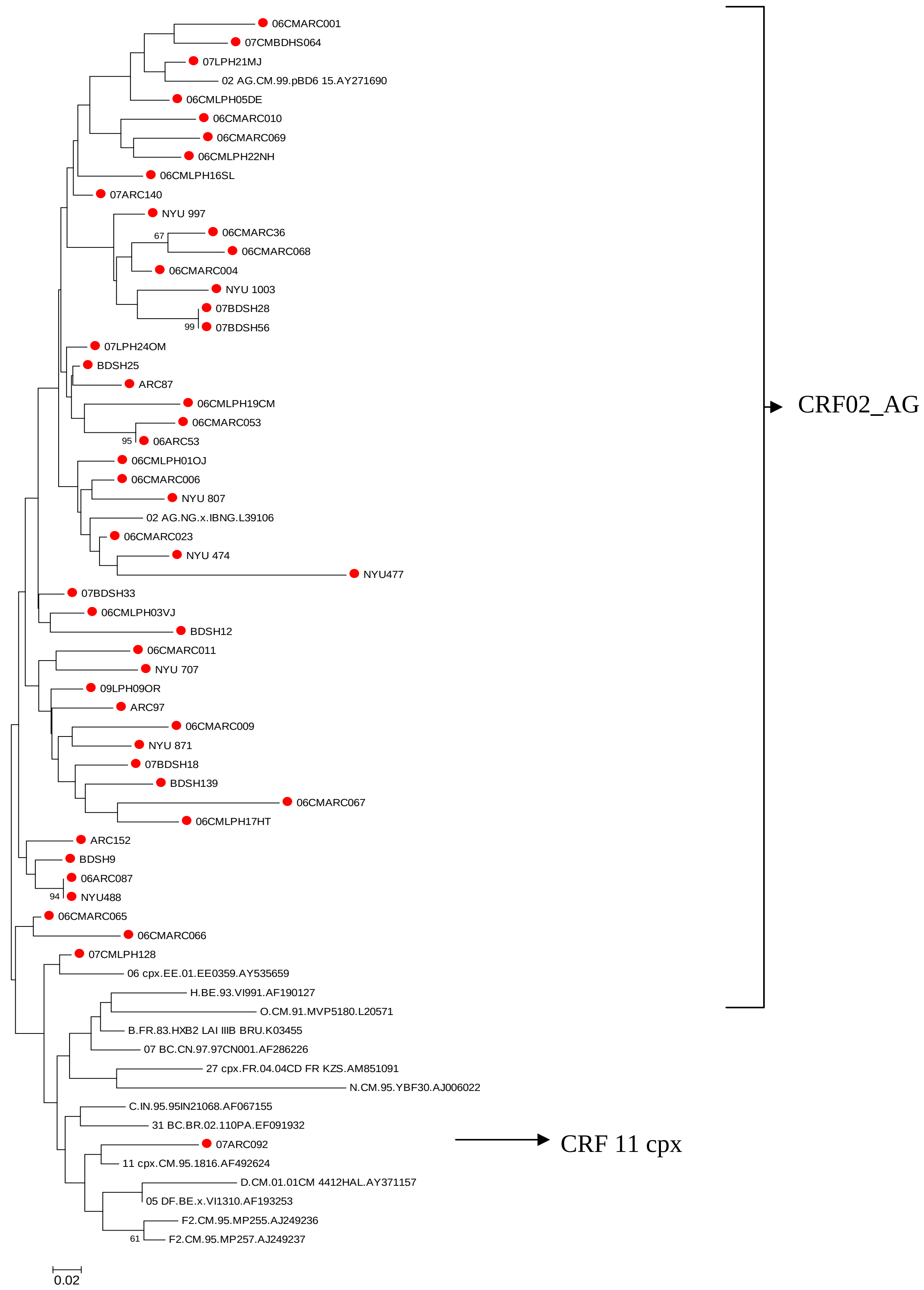
06CMARC001
07CMBDHS064
07LPH21MJ
02 AG.CM.99.pBD6 15.AY271690
06CMLPH05DE
06CMARC010
06CMARC069
06CMLPH22NH
06CMLPH16SL
07ARC140
NYU 997
67
06CMARC36
06CMARC068
06CMARC004
NYU 1003
07BDSH28
99
07BDSH56
07LPH24OM
BDSH25
ARC87
06CMLPH19CM
06CMARC053
95
06ARC53
06CMLPH01OJ
06CMARC006
NYU 807
02 AG.NG.x.IBNG.L39106
06CMARC023
NYU 474
NYU477
07BDSH33
06CMLPH03VJ
BDSH12
06CMARC011
NYU 707
09LPH09OR
ARC97
06CMARC009
NYU 871
07BDSH18
BDSH139
06CMARC067
06CMLPH17HT
ARC152
BDSH9
06ARC087
94
NYU488
06CMARC065
06CMARC066
07CMLPH128
06 cpx.EE.01.EE0359.AY535659
H.BE.93.VI991.AF190127
O.CM.91.MVP5180.L20571
B.FR.83.HXB2 LAI IIIB BRU.K03455
07 BC.CN.97.97CN001.AF286226
27 cpx.FR.04.04CD FR KZS.AM851091
N.CM.95.YBF30.AJ006022
C.IN.95.95IN21068.AF067155
31 BC.BR.02.110PA.EF091932
07ARC092
11 cpx.CM.95.1816.AF492624
D.CM.01.01CM 4412HAL.AY371157
05 DF.BE.x.VI1310.AF193253
F2.CM.95.MP255.AJ249236
61
F2.CM.95.MP257.AJ249237
CRF02_AG
CRF 11 cpx
0.02

Fig 2a2,

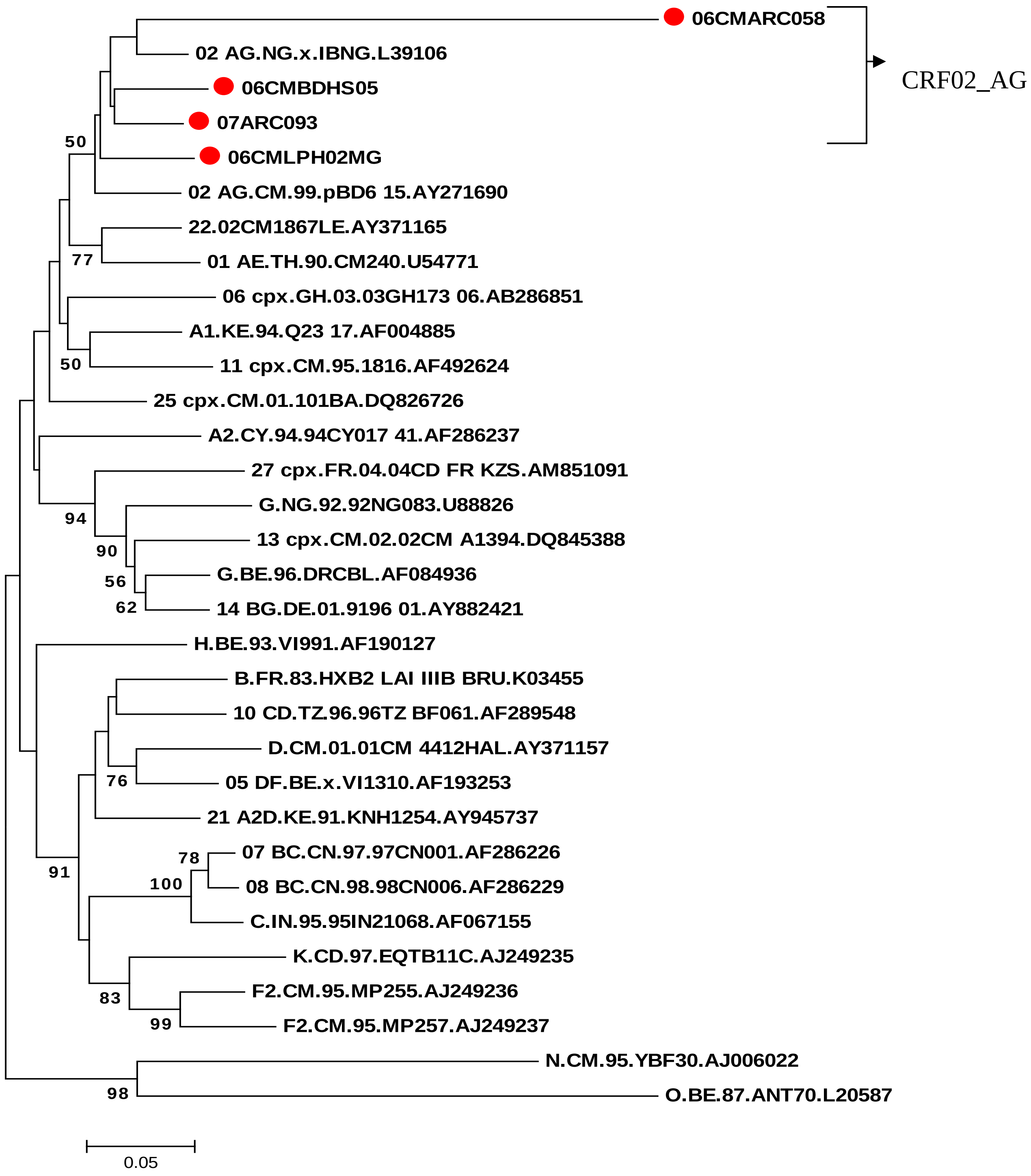

Fig 2a3

Fig 2a1,2a2,2a3 are Phylogenetic neighbor-joining tree of partial gag (p17), Patient isolates were placed after red dot. Bootstrap value of 60% and above were shown. The Subtype with which Cameroon viruses clustered was indicated in the right side with corresponding arrow mark.

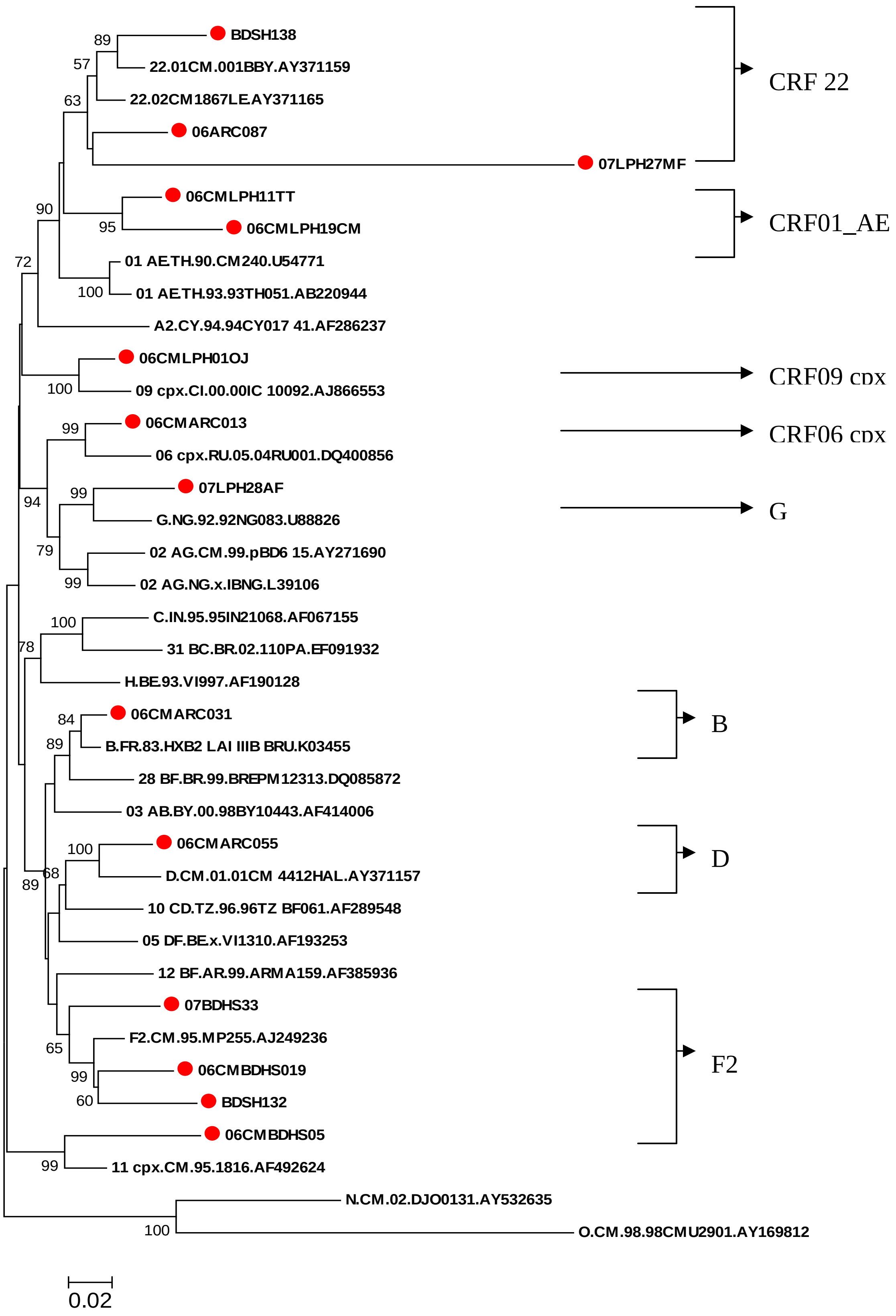

89
BDSH138
57
22.01CM.001BBY.AY371159
63
22.02CM1867LE.AY371165
06ARC087
07LPH27MF
CRF 22
90
06CMLPH11TT
95
06CMLPH19CM
CRF01_AE
72
01 AE.TH.90.CM240.U54771
100
01 AE.TH.93.93TH051.AB220944
A2.CY.94.94CY017 41.AF286237
06CMLPH01OJ
100
09 cpx.CI.00.00IC 10092.AJ866553
CRF09 cpx
99
06CMARC013
06 cpx.RU.05.04RU001.DQ400856
CRF06 cpx
99
07LPH28AF
94
G.NG.92.92NG083.U88826
G
79
02 AG.CM.99.pBD6 15.AY271690
99
02 AG.NG.x.IBNG.L39106
100
C.IN.95.95IN21068.AF067155
78
31 BC.BR.02.110PA.EF091932
H.BE.93.VI997.AF190128
84
06CMARC031
89
B.FR.83.HXB2 LAI IIIB BRU.K03455
B
28 BF.BR.99.BREPM12313.DQ085872
03 AB.BY.00.98BY10443.AF414006
100
06CMARC055
68
D.CM.01.01CM 4412HAL.AY371157
D
89
10 CD.TZ.96.96TZ BF061.AF289548
05 DF.BE.x.VI1310.AF193253
12 BF.AR.99.ARMA159.AF385936
07BDHS33
65
F2.CM.95.MP255.AJ249236
99
06CMBDHS019
60
BDSH132
F2
06CMBDHS05
99
11 cpx.CM.95.1816.AF492624
N.CM.02.DJO0131.AY532635
100
O.CM.98.98CMU2901.AY169812
0.02

Fig 2b1

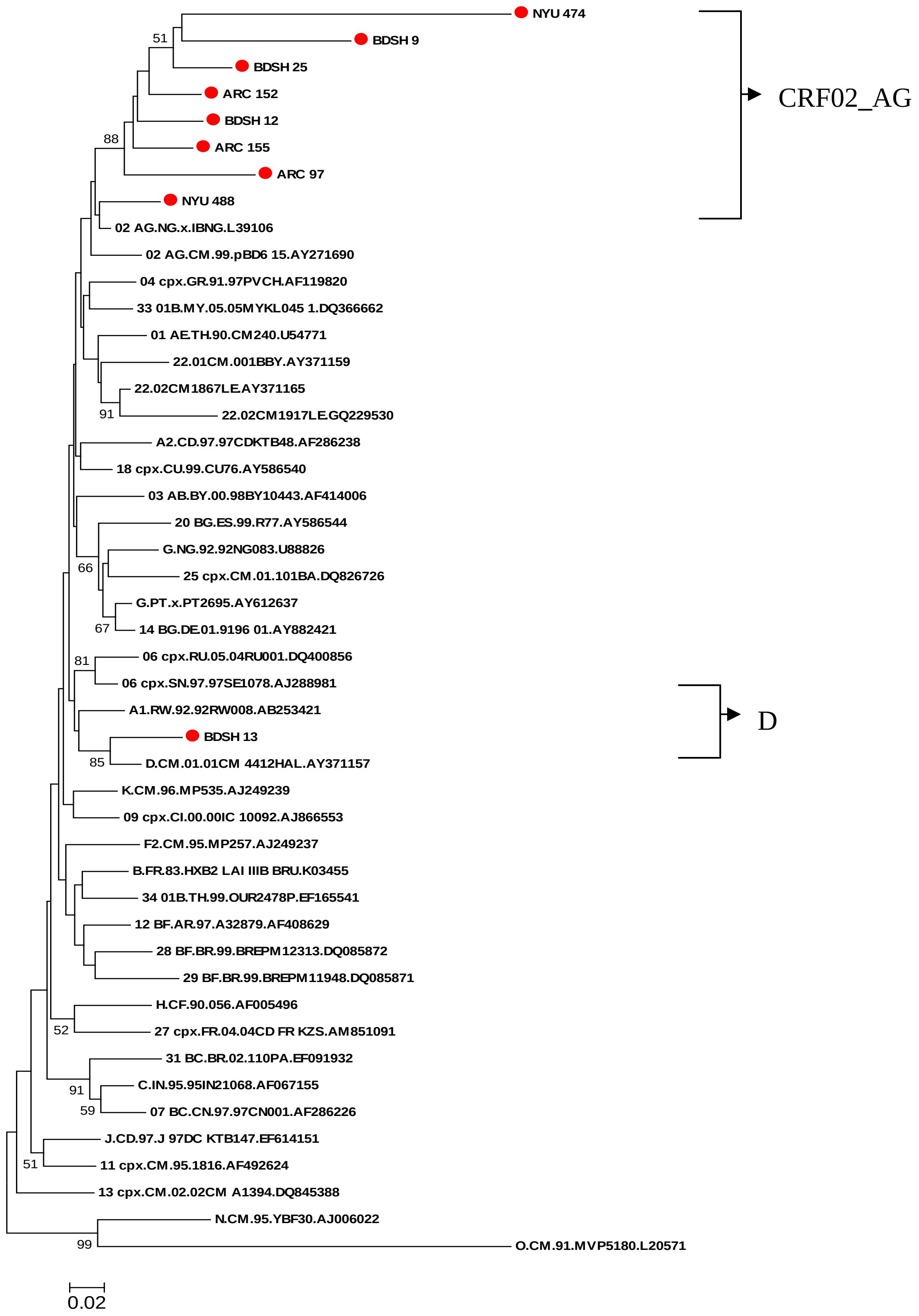
NYU 474
BDSH 9
51
BDSH 25
ARC 152
BDSH 12
88
ARC 155
ARC 97
NYU 488
02 AG.NG.x.IBNG.L39106
02 AG.CM.99.pBD6 15.AY271690
04 cpx.GR.91.97PVCH.AF119820
33 01B.MY.05.05MYKL045 1.DQ366662
01 AE.TH.90.CM240.U54771
22.01CM.001BBY.AY371159
22.02CM1867LE.AY371165
91
22.02CM1917LE.GQ229530
A2.CD.97.97CDKTB48.AF286238
18 cpx.CU.99.CU76.AY586540
03 AB.BY.00.98BY10443.AF414006
20 BG.ES.99.R77.AY586544
G.NG.92.92NG083.U88826
66
25 cpx.CM.01.101BA.DQ826726
G.PT.x.PT2695.AY612637
67
14 BG.DE.01.9196 01.AY882421
81
06 cpx.RU.05.04RU001.DQ400856
06 cpx.SN.97.97SE1078.AJ288981
A1.RW.92.92RW008.AB253421
BDSH 13
85
D.CM.01.01CM 4412HAL.AY371157
K.CM.96.MP535.AJ249239
09 cpx.CI.00.00IC 10092.AJ866553
F2.CM.95.MP257.AJ249237
B.FR.83.HXB2 LAI IIIB BRU.K03455
34 01B.TH.99.OUR2478P.EF165541
12 BF.AR.97.A32879.AF408629
28 BF.BR.99.BREPM12313.DQ085872
29 BF.BR.99.BREPM11948.DQ085871
H.CF.90.056.AF005496
52
27 cpx.FR.04.04CD FR KZS.AM851091
31 BC.BR.02.110PA.EF091932
91
C.IN.95.95IN21068.AF067155
59
07 BC.CN.97.97CN001.AF286226
J.CD.97.J 97DC KTB147.EF614151
51
11 cpx.CM.95.1816.AF492624
13 cpx.CM.02.02CM A1394.DQ845388
N.CM.95.YBF30.AJ006022
99
O.CM.91.MVP5180.L20571
0.02
CRF02_AG
D

Fig 2b2

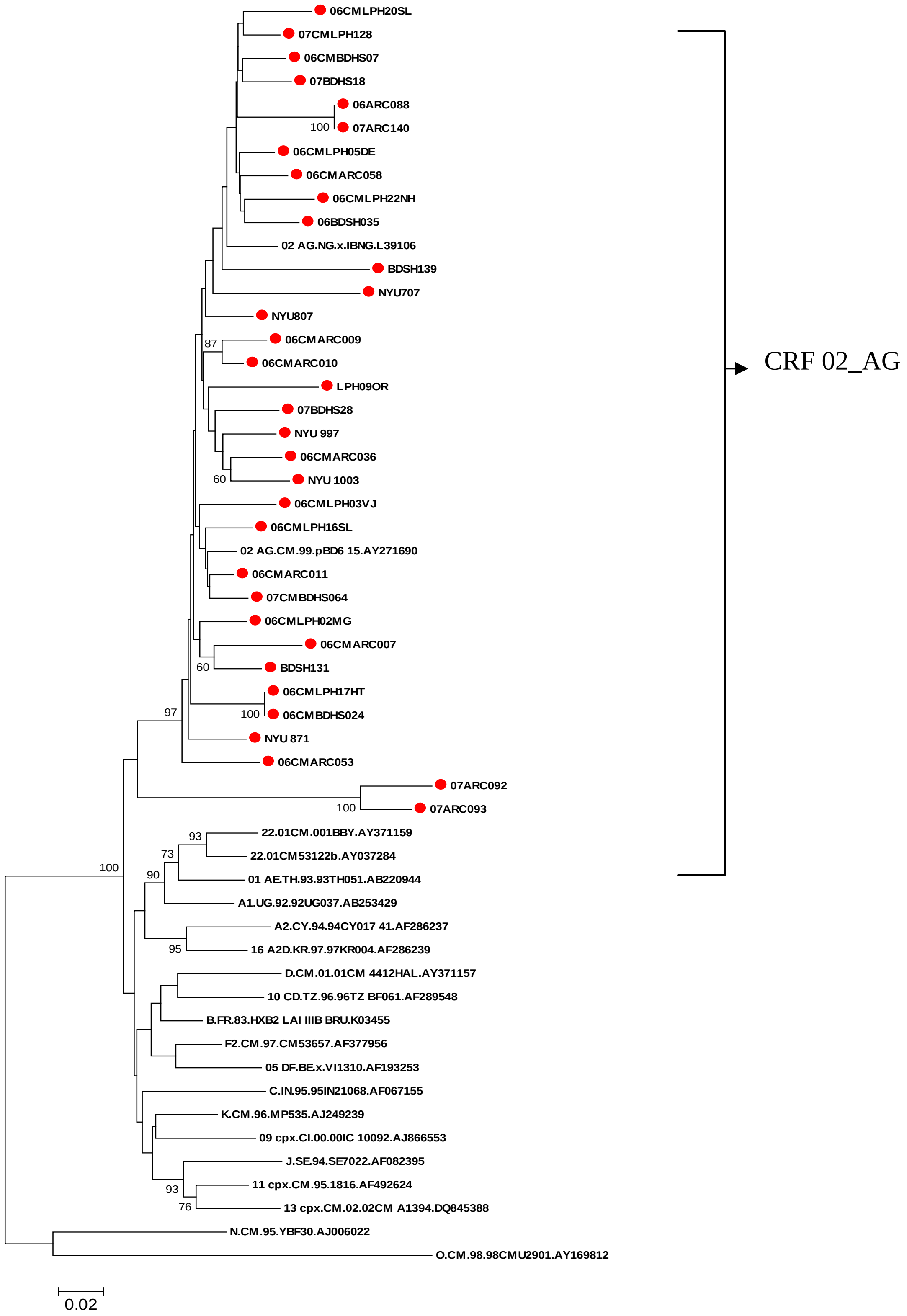

Fig 2b3

Fig 2b1-3 ***are Phylogenetic neighbor-joining tree of partial pol sequences, Patient isolates were placed after red dot. Bootstrap value of 60% and above were shown. The Subtype with which Cameroon viruses clustered was indicated in the right side with corresponding arrow mark.***

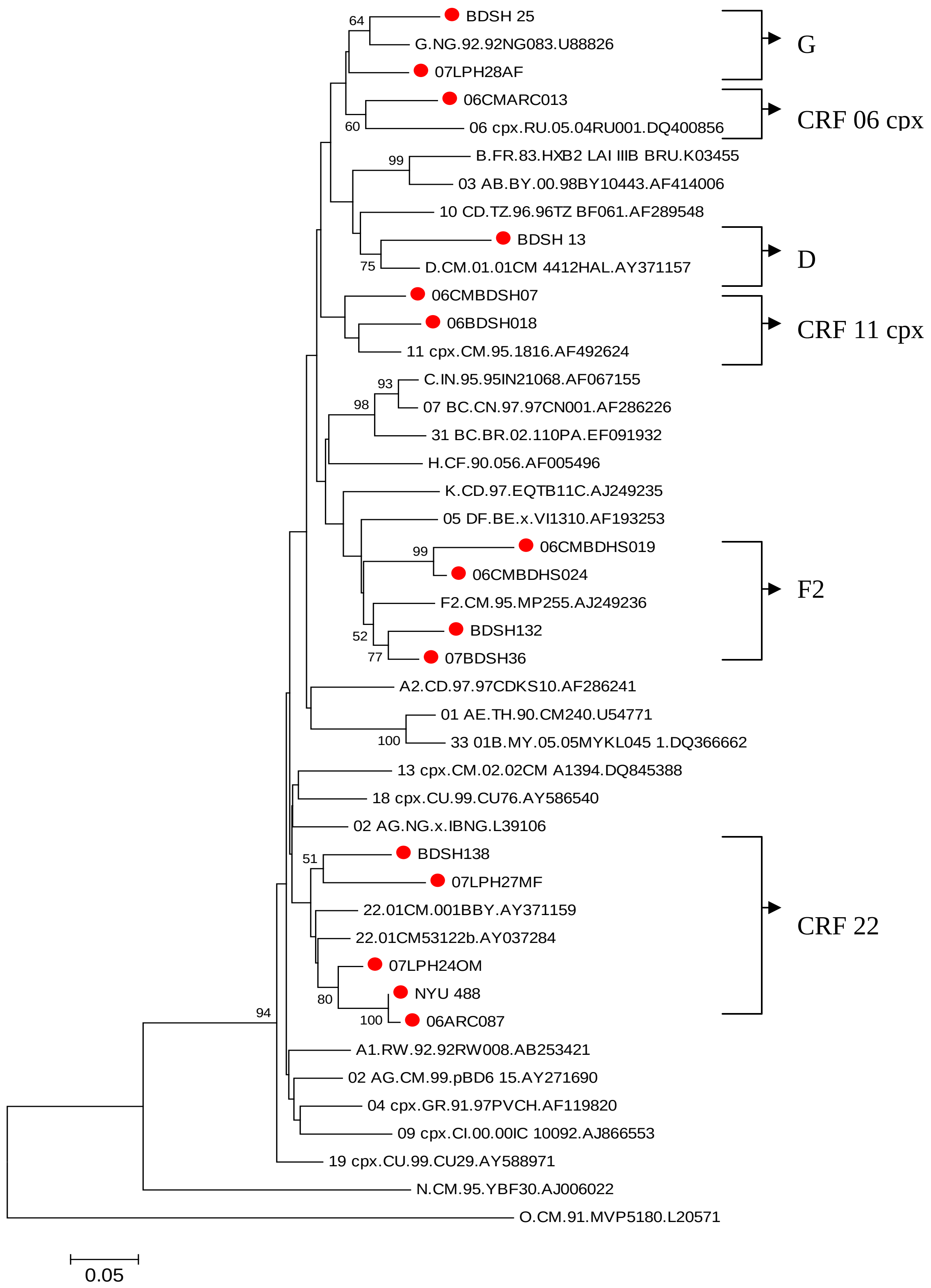
BDSH 25
G.NG.92.92NG083.U88826
07LPH28AF
06CMARC013
06 cpx.RU.05.04RU001.DQ400856
B.FR.83.HXB2 LAI IIIB BRU.K03455
03 AB.BY.00.98BY10443.AF414006
10 CD.TZ.96.96TZ BF061.AF289548
BDSH 13
D.CM.01.01CM 4412HAL.AY371157
06CMBDSH07
06BDSH018
11 cpx.CM.95.1816.AF492624
C.IN.95.95IN21068.AF067155
07 BC.CN.97.97CN001.AF286226
31 BC.BR.02.110PA.EF091932
H.CF.90.056.AF005496
K.CD.97.EQTB11C.AJ249235
05 DF.BE.x.VI1310.AF193253
06CMBDHS019
06CMBDHS024
F2.CM.95.MP255.AJ249236
BDSH132
07BDSH36
A2.CD.97.97CDKS10.AF286241
01 AE.TH.90.CM240.U54771
33 01B.MY.05.05MYKL045 1.DQ366662
13 cpx.CM.02.02CM A1394.DQ845388
18 cpx.CU.99.CU76.AY586540
02 AG.NG.x.IBNG.L39106
BDSH138
07LPH27MF
22.01CM.001BBY.AY371159
22.01CM53122b.AY037284
07LPH24OM
NYU 488
06ARC087
A1.RW.92.92RW008.AB253421
02 AG.CM.99.pBD6 15.AY271690
04 cpx.GR.91.97PVCH.AF119820
09 cpx.CI.00.00IC 10092.AJ866553
19 cpx.CU.99.CU29.AY588971
N.CM.95.YBF30.AJ006022
O.CM.91.MVP5180.L20571
64
60
99
75
93
98
99
52
77
100
51
80
100
94
G
CRF 06 cpx
D
CRF 11 cpx
F2
CRF 22
0.05

Fig 2c1

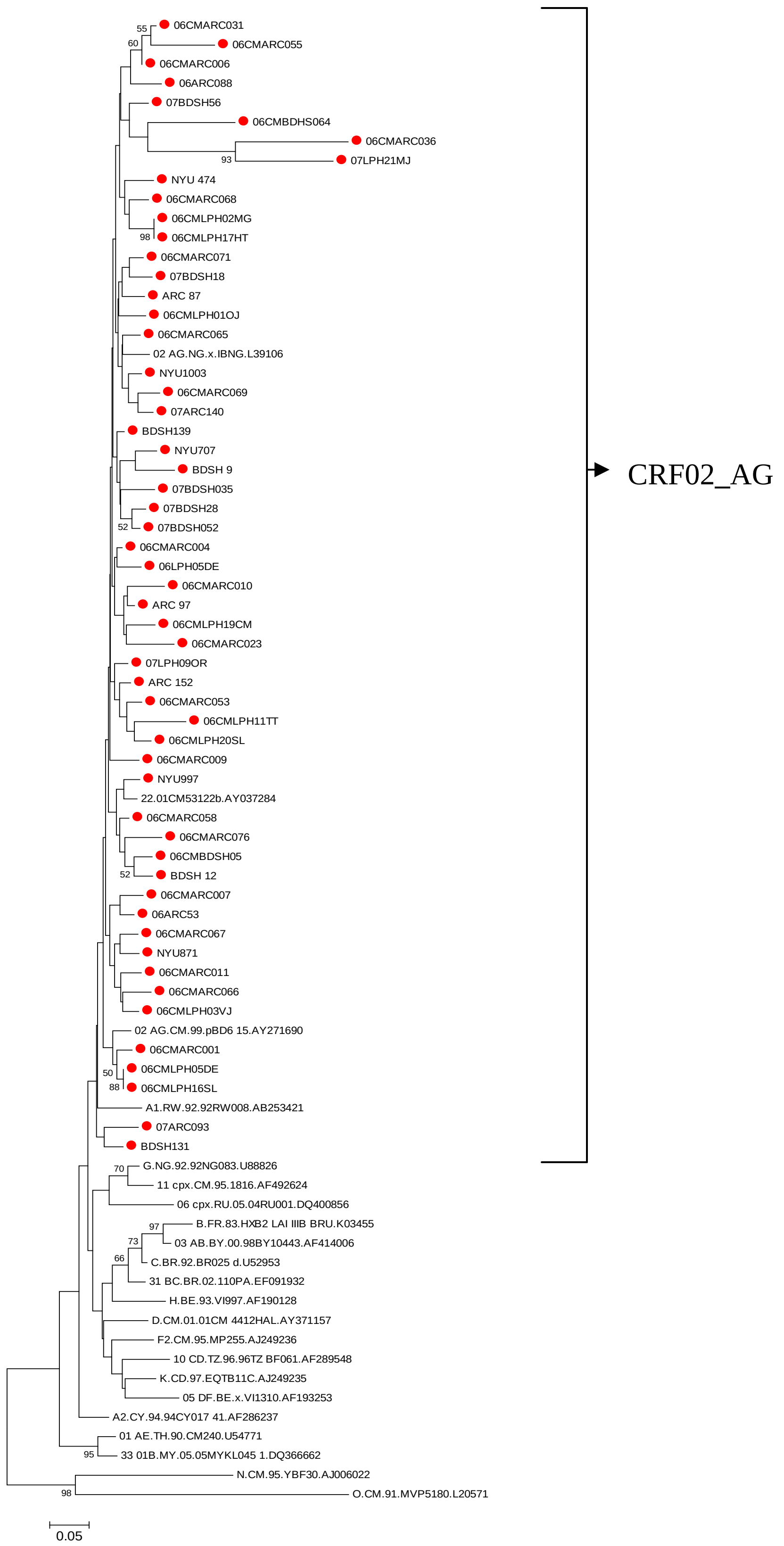
06CMARC031
06CMARC055
06CMARC006
06ARC088
07BDSH56
06CMBDHS064
06CMARC036
07LPH21MJ
NYU 474
06CMARC068
06CMLPH02MG
06CMLPH17HT
06CMARC071
07BDSH18
ARC 87
06CMLPH01OJ
06CMARC065
02 AG.NG.x.IBNG.L39106
NYU1003
06CMARC069
07ARC140
BDSH139
NYU707
BDSH 9
07BDSH035
07BDSH28
07BDSH052
06CMARC004
06LPH05DE
06CMARC010
ARC 97
06CMLPH19CM
06CMARC023
07LPH09OR
ARC 152
06CMARC053
06CMLPH11TT
06CMLPH20SL
06CMARC009
NYU997
22.01CM53122b.AY037284
06CMARC058
06CMARC076
06CMBDSH05
BDSH 12
06CMARC007
06ARC53
06CMARC067
NYU871
06CMARC011
06CMARC066
06CMLPH03VJ
02 AG.CM.99.pBD6 15.AY271690
06CMARC001
06CMLPH05DE
06CMLPH16SL
A1.RW.92.92RW008.AB253421
07ARC093
BDSH131
G.NG.92.92NG083.U88826
11 cpx.CM.95.1816.AF492624
06 cpx.RU.05.04RU001.DQ400856
B.FR.83.HXB2 LAI IIIB BRU.K03455
03 AB.BY.00.98BY10443.AF414006
C.BR.92.BR025 d.U52953
31 BC.BR.02.110PA.EF091932
H.BE.93.VI997.AF190128
D.CM.01.01CM 4412HAL.AY371157
F2.CM.95.MP255.AJ249236
10 CD.TZ.96.96TZ BF061.AF289548
K.CD.97.EQTB11C.AJ249235
05 DF.BE.x.VI1310.AF193253
A2.CY.94.94CY017 41.AF286237
01 AE.TH.90.CM240.U54771
33 01B.MY.05.05MYKL045 1.DQ366662
N.CM.95.YBF30.AJ006022
O.CM.91.MVP5180.L20571
55
60
93
98
52
52
50
88
70
97
73
66
95
98
0.05
CRF02_AG

Fig 2c2

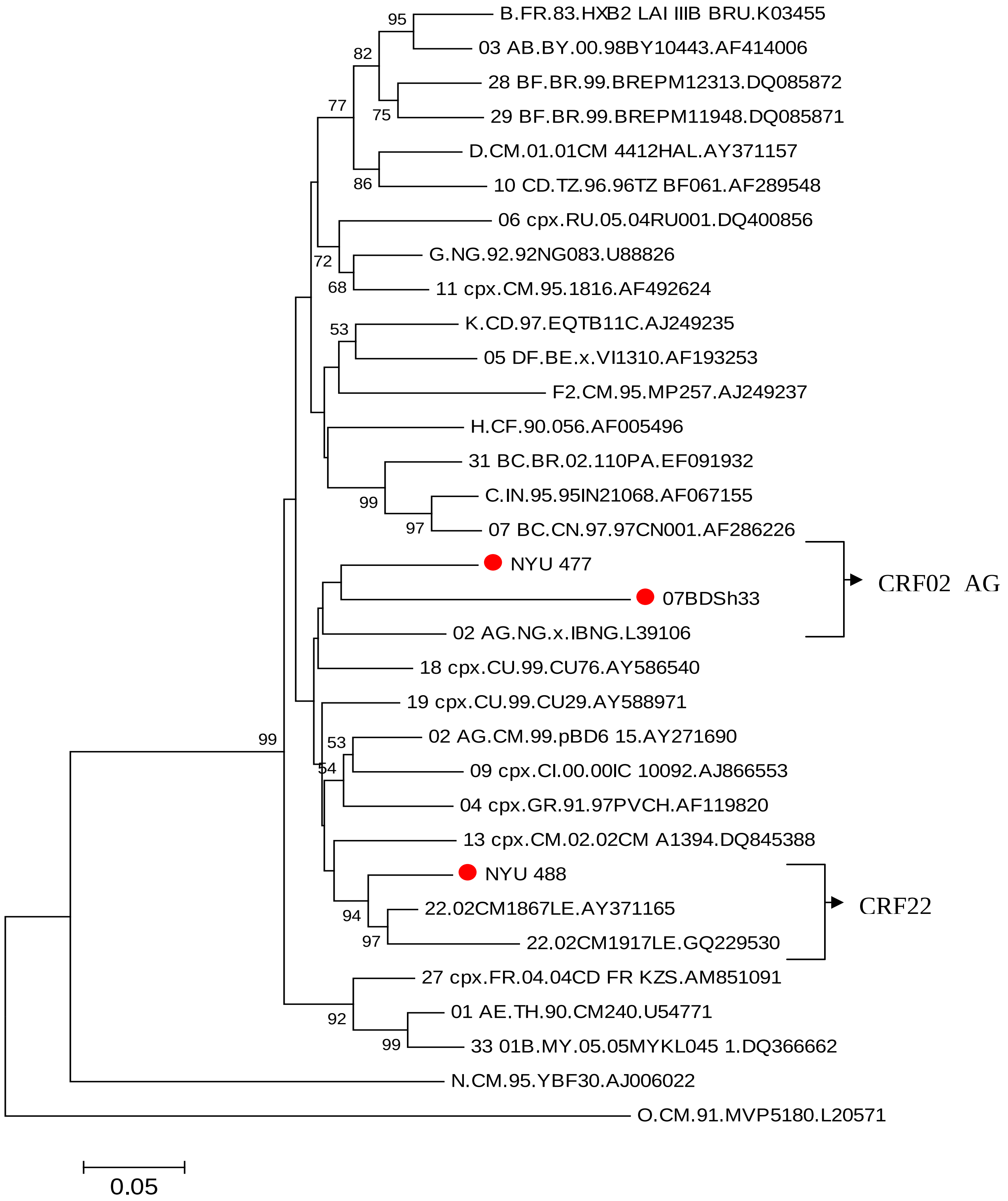

Fig 2c1

Fig 2c1-3 ***are Phylogenetic neighbor-joining tree of partial Env (gp41) sequences, Patient isolates were placed after red dot. Bootstrap value of 60% and above were shown. The Subtype with which Cameroon viruses clustered was indicated in the right side with corresponding arrow mark.***
